# Recommendations for a Complete Reporting of Statistical Methods in Veterinary Pharmacology

**DOI:** 10.1111/jvp.70001

**Published:** 2025-06-06

**Authors:** Nicolas F. Villarino

**Affiliations:** ^1^ Department of Veterinary Clinical Sciences College of Veterinary Medicine, Washington State University Pullman WA USA

**Keywords:** assumptions, experimentation, replicability, reproducibility, statistics

## Abstract

Reproducibility and replicability of study results are crucial for advancing scientific knowledge. However, achieving these goals is often challenging, which can compromise the credibility of research and incur immeasurable costs for the progression of science. Despite efforts to standardize reporting with guidelines, the description of statistical methodology in manuscripts often remains insufficient, limiting the possibility of replicating scientific studies. A thorough, transparent, and complete report of statistical methods is essential for understanding study results and mimicking statistical strategies implemented in previous studies. This review outlines the key statistical reporting elements required to replicate statistical methods in most current veterinary pharmacology studies. It also offers a protocol for statistical reporting to aid in manuscript preparation and to assist trialists and editors in the collective strive for advancing veterinary pharmacology research.

## 1 Introduction

The complete and accurate reporting of statistical methods is imperative for understanding study results and emulating the statistical methodology implemented for the data analysis in previous studies.

The ability to repeat specific statistical procedures to analyze scientific data contributes to the reproducibility and replicability of study results. Study results are reproducible when a researcher produces similar results by applying the same methods to the same scientific question and data; for example, by analyzing data available in data repositories. Study results are considered replicable when a researcher produces similar results by applying the same methods to the same scientific question but with new data; for example, by analyzing data from new experiments. It is widely recognized that scientific research that cannot be reproduced or replicated can hinder scientific progress (Ioannidis 2005; Munafò et al. 2017; Goodman et al. 2016). In the field of preclinical biomedical research, the lack of reproducibility has been a significant concern in recent years. A study found that 70% of researchers (out of 1500 scientists) had attempted and failed to reproduce another scientist's experiments (Baker 2016b). It is not known whether there is a significant number of irreproducible studies in veterinary pharmacology, but it is clear that this issue should also be addressed in this field.

Incomplete reporting of statistical methods in veterinary pharmacology studies presents a significant challenge for reproducing and replicating scientific studies, like in any other field (Gosselin 2021). It is common for the statistical sections of these studies to omit important information needed to mimic data analysis strategies, such as underlying statistical test assumptions, effect size used in sample size calculation, and statistical diagnostics.

Improving reporting standards by including a complete statistical methodology would help increase the likelihood of publishing reproducible and replicable studies and advance the veterinary pharmacology field. This study outlines the essential elements for recapitulating statistical strategies in current veterinary pharmacology studies. It also reviews statistical test assumptions and provides a protocol for statistical reporting to aid in manuscript preparation and assist trialists and editors in evaluating the appropriateness of data analysis.

## 2 Study Design Section

The selection of statistical strategies—comprising the tools and methodologies employed in the study—is crucially influenced by the experimental design and the underlying scientific and statistical hypotheses. Authors and manuscript reviewers need to prioritize the clarity of the statistical approach, ensuring that it is not only thoroughly articulated but also perfectly aligned with the scientific and statistical hypotheses, study design, and overarching conclusions. Clear and precise reporting in this area strengthens the integrity and impact of research findings.

### 2.1 Scientific and Statistical Hypotheses

The scientific hypothesis should contain key elements for deriving the statistical hypothesis and framing the experimental design. A statistical hypothesis is a scientific question expressed in terms of statistical parameters; for example, the μ = zero or the μ_treatment A_ = μ_treatment B_. Unfortunately, statistical questions are rare in pharmacology manuscripts. Including statistical questions can greatly facilitate the evaluation of the statistical strategies utilized, ultimately resulting in more reliable and credible research findings.

A statistical hypothesis is deconstructed from the scientific hypotheses (Hand 1994). The scientific and statistical hypotheses inform the statistical strategy, for example, selecting one‐ or two‐sided statistical testing. Therefore, if the statistical question is not presented explicitly in the manuscript, it needs to be conceptualized by the article reader to understand the rationale behind the statistical strategy. The incorrect description or interpretation of the scientific hypothesis can result in an inappropriate statistical strategy and study conclusions. The PICOT format is a helpful approach for framing research questions to facilitate establishing the statistical hypothesis (Table 1) (Guyatt et al. 2008).

**TABLE 1:** The PICOT format for framing research questions that explore the effect of therapy (updated from Guyatt et al. 2008).

PICOT format		Example
(P)	Population refers to the sample of experimental units to recruit	Cats diagnosed with chronic kidney disease admitted to the Veterinary Teaching Hospital at Washington State University
(I)	Intervention refers to the treatment that will be administered to the enrolled experimental units	Administration of meloxicam every 24 h
(C)	Comparison: It refers to a negative, positive, sham, and control groups	Administration of buprenorphine every 24 h
(O)	Outcome represents the outcome primary and or secondary variable(s) to be assessed.	Serum creatinine concentration (primary outcome), systemic blood pressure (secondary outcome)
(T)	Time describes the duration of your data collection	Cats will be followed over 1 week

*Note:* The administration of meloxicam every 24 h for a week to adult cats diagnosed with chronic kidney disease admitted to the Veterinary Teaching Hospital at Washington State University increases serum creatinine concentrations and systemic blood pressure above the normal reference values faster than in cats receiving buprenorphine every 24 h.

### 2.2 Study Design Considerations

The effectiveness of statistical tools and methodologies is intrinsically tied to the nuances of study design. Factors such as covariates, randomization, sampling methods, treatment design, and research assumptions are important in shaping the analytical approach. Additionally, the nature of the experimental units—individual or pen of animals—must be carefully considered. Strategies for controlling bias and error, along with the types of data measurement scales used, further influence the statistical strategy. Thus, it is clear that a successful study design cannot exist in isolation from a thoughtful statistical strategy. Recognizing this interdependence is vital for achieving credible and impactful research outcomes.

Statisticians are trained to picture the study design–statistical strategy interdependence. Engaging a statistician during the early stages of a study is crucial for optimizing its design and maximizing the design of powerful statistical strategies before implementation. A prime example of how overlooking this step can lead to study flaws is the choice of a crossover design over a parallel design when there are potential unequal carryover effects. This oversight can significantly compromise the study's integrity (*vide infra*). Regrettably, many studies omit statisticians from the design process, which can lead to serious ramifications. The burden of ensuring the manuscript's quality often falls on reviewers, who must be vigilant in identifying incomplete reporting and experimental flaws. These incomplete reports hinder the readers' ability to evaluate the study effectively and jeopardize trust in whether the statistical strategies employed truly address the scientific hypothesis and support the study's conclusions.

### 2.3 Reporting Sample Size Justification and Calculation

The importance of the sample size calculation and statistical power is ubiquitous when conducting frequentist‐based analysis (Guo et al. 2013; Guo and Pandis 2015; Liu and Liang 1997). The optimal number of experimental units must balance between inadequate statistical power and accurate representation of the population of interest (Krzywinski and Altman 2013). While a small sample size may result in Type II statistical error, an excessively large sample size for maximizing the study power can increase the likelihood of rejecting the null hypothesis but at the expense of detecting a trivial treatment effect (Cohen 1988; Curtis et al. 2022). Authors must clearly and transparently explain the methods used to calculate sample size. Many authors often inform only that “the sample size calculation was determined using an 80% power at α = 0.05”. This information is not enough to recapitulate sample size calculations. In addition to the significance level and statistical power, the effect size and dispersion of the outcome variable factored into the calculation are necessary to reiterate sample size calculations (Charan and Kantharia 2013; Cohen 1988; Cohen 1992). Effect size is the magnitude of a treatment effect or association of variables. There are multiple accepted effect sizes, and their selection depends on the research question and the experimental design. Regardless of the type of effect size used in the study, it should be biological or clinically relevant. In the case of studies including multiple outcome variables, it is also essential to report and justify which outcome variable was used for sample size calculations.

It is not uncommon to calculate sample sizes by increasing or decreasing the effect size based on the available budget or the availability of a “convenient” or “practical” number of experimental units rather than on the scientific relevance of the effect size. This practice is mathematically possible but goes against the essence of frequentist statistical analysis and sample size calculation. Ironically, scientists are reluctant to determine a sample size or use an inadequate method for calculating it, increasing the likelihood that their study result is a Type II statistical error or, more commonly, a Type I statistical error. The sequential scientific approach to estimate the sample size for a study should first determine the clinically or biologically relevant effect size; second, define the expected variability of the outcome variable in the population, and then establish the desired statistical power and alpha.

If applicable, acknowledgment of the number of independent replications for each experiment is also necessary. Well‐designed studies are expected to have experimental groups with an equal number of experimental units. However, an unequal sample size is acceptable for some experimental designs, such as in matching case–control studies (Bate and Karp 2014). The inclusion of unequal sample sizes needs to be justified. The software used to calculate sample size calculations should also be listed.

### 2.4 Reporting Randomization of Experimental Units

Randomizing experimental units into treatment groups is considered good statistical practice for controlling bias and maximizing the appropriate comparability of experimental groups (Althouse et al. 2021). Without randomization, differences between treatment and control groups may not be solely attributed to the treatment.

Randomization of experimental units is a formal process. It is not just someone separating animals into experimental groups on a whim. Detailing the randomization method, including any restrictions, such as blocking or stratification, and allocation ratio, is required (Althouse et al. 2021). It is also important to report who generated the allocation sequence, enrolled study units, and assigned units to their respective groups. When randomization is not feasible, a valid scientific justification must be provided.

The comparison of baseline variables between treatment groups is a contentious subject when it comes to statistical significance tests. The readers are encouraged to review the seminal studies on this topic authored by Altman 1985; Altman and Doré 1990; Begg 1990; Senn 1991, 1994, and CONSORT (Butcher et al. 2022).

## 3 Data Analysis Section

### 3.1 Masking Data Collection and Analysis

Masking is a minimum requirement in experimental designs to control bias (Kaptchuk 1998). Masking the statistician can help to control bias (Boutron et al. 2006; Collins et al. 2020). Researchers should explicitly state whether a study was masked, who was blinded, how blinding was achieved, and the reasons for unplanned unmasking. In some studies, masking is impossible (Anand et al. 2020). If it is impossible to mask the statistician, or it needs to be unblinded at some stages of the data analysis (e.g., for interim or safety analysis) (Iflaifel et al. 2023), a valid scientific justification should be provided.

### 3.2 Reporting Data Handling Strategies

Any data transformation or scaling needs to be acknowledged to maximize the reproducibility of the statistical analysis. An outlier is an extreme observation inconsistent with the data's main body. Outliers can modify the data distribution (e.g., from normal to non‐normal distribution) (Zimmerman 1994). Interpreting transformed data can be an obstacle, especially if complex transformations or a combination of transformations are implemented. Log‐transformed data need to be back‐transformed to be expressed on the original scale of the measurement. Instead, this back‐transformed mean is the geometric mean, and confidence intervals are not symmetrical (Bland and Altman 1996). If the data were normalized, it is necessary to define the meaning for 100% and 0%.

An infinite number of things can go wrong in a study, so achieving an unblemished data set is rare. Outliers' handling strategies need to be reported judiciously and openly. Reporting the values of removed data, if any, along with the entire raw dataset, is good statistical practice and may be valuable information for other researchers. Attrition/censoring, truncation, and/or the exclusion of study subjects are other factors that must be thoroughly addressed to facilitate replication of the statistical methods implemented (Charan and Kantharia 2013). Missing data are common and pose considerable challenges in the analyses and interpretation of clinical research (Little et al. 2012), as well as compromise inferences of clinical trials (Yeatts and Martin 2015). The performance of various statistical tests can be significantly impacted by unbalanced data, particularly paired testing, such as the paired Student's t‐test. Missing, uninterpretable, or equivocal data should be described. It is necessary to clearly report whether the data loss affected the sample size in the study's methods section. Also, it is essential to provide detailed explanations of the procedures for replacing experimental units, including randomization strategies. Additionally, it is imperative to address why replacements were not feasible and the approach for handling missing data during the analytical phase (Li et al. 2015; Perkins et al. 2018). Unequal numbers of experimental units may require a statistical correction, particularly if parametric testing is implemented (Welch 1947).

Furthermore, when reporting the sample size, it is vital to specify what has been counted, such as technical replicates or repeat experiments. The section should accurately describe each data set's group size (n), emphasizing that “n” refers to independent values, not replicates (Curtis et al. 2022).

### 3.3 Reporting Statistical Models

Researchers should avoid, and reviewers should be vigilant about incorrect model selection, such as using unpaired methods for paired data for comparing survival times or the Chi‐square test rather than Fisher's Exact Test when low cell frequencies are available (Šimundić and Nikolac 2009). Regarding the use of covariates, reviewers should determine if the covariates adjusted for in models are appropriate. A priori identification of covariates used for adjustment is preferable; a priori identification of covariate selection based on univariate analyses is generally discouraged. Also, if the study includes hierarchical data structures (e.g., cluster randomized trials, repeated measures, or matching of cases and controls), it is important to describe how these data structures have been analyzed.

Veterinary pharmacology studies often implement repeated measurements of the dependent variable (s) collected at several time points. Data collected repetitively may violate the assumption of data independence of some statistical tests (such as ANOVA, within‐subject measurements correlation) and would require special models, as otherwise, it could result in a Type I statistical error.

Repeated measurements are often analyzed by implementing linear models (i.e., ANOVA‐repeated measures) or mixed linear models (Duricki et al. 2016). ANOVA‐repeated measures account for within‐subject correlations and assume that the variances of differences between treatment levels are equal (an assumption known as sphericity). This assumption can be checked by Mauchly's test (Mauchly 1940). If this assumption is not met, Greenhouse–Geisser correction or Huynh–Feldt correction adjustments are used to correct violations of the sphericity assumption (Greenhouse and Geisser 1959; Huynh and Mandeville 1979).

In contrast, mixed linear models handle within‐subject correlations over time and data with nonconstant variability. This approach can handle balanced as well as unbalanced or missing within‐subject data. Mixed linear models are constructed based on the within‐subject covariance structures that handle within‐subject correlations. Models ignoring the within‐subject correlation by using a suboptimal covariance structure will increase the Type I or II statistical error rate for fixed effect tests in the analysis. There are multiple covariance structures that can be used to account for the within‐subject correlations over time and data with nonconstant variability, such as the first‐order autoregressive covariance structure, first‐order ante dependence covariance, etc. (Littell et al. 2000). Importantly, each covariance structure may have its own requirements. For example, the first‐order autoregressive covariance structure requires equally spaced times (Littell et al. 2000). Selection of the best simplest model is based on a comparison of the model fitting statistics information, such as the Akaike Information Criteria, the finite‐sample corrected Akaike Information Criteria, and Schwarz's Bayesian Information Criteria (Heo et al. 2020). When the mixed model approach is used, the report should describe which model terms were considered fixed and which were considered random, if any. Of course, it should specify which variance–covariance structure was used.

For reproducibility of the statistical modeling, reporting diagnostics (such as sum of squared errors, R^2^, adjusted R^2^, Akaike information criteria, etc.) as well as a summary model specification (e.g., goodness‐of‐fit) test are helpful for emulating or approximating the final model performance. When applicable, mention the one‐sidedness of the test if a one‐sided test is used and explain precisely which model was fitted to the data and whether (and how) data were weighted (e.g., for a curve using nonlinear regression). In the case of multilinear regression analysis, the strategies for the selection of explanatory variables (e.g., backward and forward selection) in model building should be presented as part of the model selection (Heinze et al. 2018).

Bayesian analysis is likely to gain more relevance in the analysis of veterinary pharmacology data (Woodward 2024). This approach provides a flexible framework that requires the translation of subjective prior beliefs into a mathematically formulated prior and the use of simulation methods, underscoring the importance of a comprehensive report of the statistical methodology implemented. Bayesian analysis produces posterior distributions that are heavily influenced by the priors. Therefore, a remarkable limitation of Bayesian analysis is the subjectivity involved in choosing a prior distribution, which can introduce bias if not carefully considered. Reports usually fail to fully report the parameter estimates assumed in the prior distribution, the reasons for the choices, the models, the sensitivity analysis, Markov chain Monte Carlo convergence measures, and the computer code. Specific reporting recommendations have been described comprehensively by Kruschke (2021) and summarized in Table 3.

Crossover study designs are extensively used in veterinary pharmacology (Mills et al. 2022; Mones et al. 2022). Analysis of variance is commonly used to test the data resulting from studies implementing a crossover experimental design (Grizzle 1965; Kenward and Jones 1987). Results from a study implementing a 2‐sequence, 2‐period crossover study design can be polluted by sequence and period effects (Senn 1988). Reports should explicitly describe the methods and results used for testing sequence and period effects. Providing a valid scientific explanation of how this was addressed is essential in the event of a sequence or period effect. The unequal carryover effect demands special consideration in two‐period crossover designs, as it can significantly impact the study's validity and may require discarding data from the second period. Reports should distinctly detail the methods and results used to confirm the absence of an unequal carryover effect (Stegemann et al. 2006). One strategy to avoid unequal carryover effects is to use an optimal washout period (Hills and Armitage 2004). A valid scientific justification for the length of the washout period should be included in study reports. Washout periods are commonly based on the plasma drug elimination half‐lives. However, the pharmacodynamic half‐life can help determine a more appropriate washout period when it exceeds the plasma drug elimination half‐life, when there is a dissociation between pharmacokinetics and pharmacodynamics (hysteresis), or when the drug's effects have a residual impact on processes influencing drug disposition (e.g., drug metabolism induction) (Hurbin et al. 2012).

### 3.4 Reporting Underlying Statistical Assumptions

Statistical tests are built upon several assumptions. Violation of tests' assumptions can result in misleading conclusions. It is not unusual to find manuscripts that fail to report whether and how the underlying statistical assumptions of the tests implemented were tested.

One of the most widely known assumptions of parametric statistics is the assumption that errors (model residuals) are normally distributed (Lumley et al. 2002). Some parametric tests also assume that the data have equal variances, which could be affected by unequal sample sizes (e.g., from unexpected dropouts of experimental units), particularly when sample sizes are relatively small (Derrick et al. 2016; Ruxton 2006; Welch 1947). A list of assumptions and recommended tests for statistical analysis commonly used in veterinary pharmacology is presented in Table 2.

**TABLE 2:** Underlying assumptions of statistical tests commonly used in veterinary pharmacology.

Test	Main underlying assumptions	Recommended test for corroborating underlying assumptions	References
**Parametric tests**			
Unpaired Student's T‐test	Each group sample is drawn from a normally distributed population.	Kolmogorov–Smirnov test, Shapiro–Wilk test, D'Agostino test. Quantile–Quantile plot of residuals	Kolmogorov (1933); Ghasemi and Zahediasl (2012); Hazelton (2003); Schucany and Ng (2006)
	Homogeneity of variance	F‐test	Moser and Stevens (1992)
	Random independent samples	Based on the experimental design Graphical data inspection Durbin–Watson for testing autocorrelation	King (1992); Durbin and Watson (1950)
Paired Student's t‐test	Each group sample is drawn from a normally distributed population.	Kolmogorov–Smirnov test, Shapiro–Wilk test, D'Agostino test. Quantile–Quantile plot of residuals	Das (2016) Hazelton (2003); Schucany and Ng (2006)
	Homogeneity of variance	F test	Moser and Stevens (1992)
	Not independent samples	Pearson correlation coefficient	Pearson (1931)
One‐way ANOVA	Each group sample is drawn from a normally distributed population	Kolmogorov–Smirnov test, Shapiro–Wilk test, D'Agostino test. Quantile–Quantile plot of residuals	Vrbik (2018) Shapiro and Wilk (1965)*;* D'Agostino and Pearson (1973)*;* Wilk and Gnanadesikan (1968); Das (2016) Hazelton (2003); Schucany and Ng (2006)
	Homogeneity of variance	Forsythe test Bartlett's test Cochran's Levene test *F*‐test	Wang et al. (2017); Bartlett (1937); Levene (1960); Cochran (1941)
	Random independent samples	Based on the experimental design Graphical data inspection Durbin–Watson for testing autocorrelation	King (1992); Durbin and Watson (1950)
ANOVA Crossover	Similar to one‐way ANOVA		
	There is no unequal carryover effect, no period effect, and no treatment‐period interactions.		Sturdevant and Lumley (2016); Cleophas (1990)
ANOVA Repeated measures *(Least Squares method)*	Similar to one‐way ANOVA		
	Sphericity (variances of the differences between all combinations of related groups are not equal)	Maulchly's test of Sphericity Greenhouse and Geisser and Huynd–Feldt Sphericity correction	Greenhouse and Geisser (1959); Mauchly (1940); Huynh and Mandeville (1979)
ANOVA Repeated measures *(Maximum likelihood method)*	Similar to one‐way ANOVA		
	Specific requirements for each covariance structure (no sphericity assumption)	Based on experimental design and comparison of the model fitting statistics information, such as Akaike Information Criteria and the Schwarz's and Bayesian Information Criteria.	Heo et al. (2020)
Regression	The errors of the model should be normally distributed (normality assumption)	Kolmogorov–Smirnov test, Shapiro–Wilk test, D'Agostino test. Quantile–Quantile plot of residuals	Vrbik (2018); Shapiro and Wilk (1965)*;* D'Agostino and Pearson (1973)*;* Wilk and Gnanadesikan (1968)*;* Das (2016); Hazelton (2003); Schucany and Ng (2006)
	The variance in the regression error “e” (or the spread of the response around the regression line) is constant across all values of the predictor X, i.e., the samples are homoscedastic.	Bartlett's and Cochran's	Bartlett (1937); Cochran (1941); White (1980)
	The dependent variable Y and the predictors should be linearly (and additively) related through the regression coefficient b.	Graphical data inspection	Williams, Grajales and Kurkiewicz (2013)
	Each value of the dependent variable Y is influenced by only a single value of the predictor X, meaning that all observations and regression errors e_i_ are independent.	Based on the experimental design	Quinn and Keough (2002)
	Multicollinearity in multilinear regression analysis	Variance inflation	Kutner et al. (2004)
Pearson product–moment correlation	The two variables (interval and ratio data) correlated are continuous	Based on the experimental design	Pearson (1932)
	The relationship between the two variables is rectilinear	Scatter plot of the two variables	
	The joint distribution of the scores is a bivariate normal distribution	Bivariate Gamma plot (chi‐squared Quantile–Quantile plot)	Johnson and Wichern (2007)
	The scores were obtained in independent pairs, each pair being unconnected from the other pairs.	Graphical data inspection Durbin–Watson for testing autocorrelation	King (1992); Durbin and Watson (1950)
**Nonparametric tests**			
Mann–Whitney U test	Similar to unpaired student tests, but no assumptions about data distribution		Fay and Proschan (2010)*;* Mann et al. (1947)
Wilcoxon‐matched pair test	Similar to the paired Student's t‐test, but no assumptions about data distribution	The pairing efficiency can be tested using Spearman correlation. It assumes that there are at least five paired measures to compare	Wilcoxon (1945)
Kruskal–Wallis test	Similar to one‐way ANOVA but with no assumptions about data distribution	See one‐way ANOVA	Kruskal and Wallis (1952)
Friedman's test	Similar to one‐way ANOVA, repeated measures but no assumptions about data distribution	See one‐way ANOVA	Friedman (1937); Friedman (1940)
Spearman correlation test	Similar to the Pearson correlation test, there are no assumptions about data distribution or a rectilinear relationship between the two variables. It can also be applied to ordinal, interval, and ratio data	See Pearson correlation test	Daniel (1990)*;* Spearman (1904)
**Other tests**			
Bonferroni correction	Individual tests are independent of each other	Based on the experimental design	Bonferroni (1936)
Benjamini–Hochberg correction	Individual tests are independent of each other Homogeneity of variances	Based on the experimental design	Benjamini and Hochberg (1995) Benjamini et al. (2001)
Dunnett's test	Homogeneity of variances	Forsythe test Bartlett's test Cochran's Levene *test*	Dunnett (2012)
Tukey's test	Homogeneity of variances and equal sample sizes	Forsythe test Bartlett's test Cochran's Levene *test*	Tukey (1949)

Authors should always report the methodology implemented to explore the data, test the underlying statistical assumptions, and acknowledge whether the assumptions have been satisfied. Manuscript reviewers are expected to be familiar with the underlying assumptions of statistical tests and should also ensure that the manuscripts include this information rather than assume that the underlying statistical assumptions have been tested and satisfied. Recapitulation of the statistical data analysis is, at least, uncertain without information about the methodology implemented for checking the underlying statistical assumptions and the assessment results.

### 3.5 Reporting Multiple Comparisons With or Without Correction

The pressure to publish scientific reports with statistically significant differences in the outcome variables often leads researchers to make questionable statistical decisions and choose methodologies that artificially create these significant results. For example, they may try multiple statistical tests or data transformation techniques until they find a statistically significant outcome. This practice is known as P‐hacking (Head et al. 2015) or data dredging (Altman and Krzywinski 2017; Ioannidis 2005) and should be avoided.

It is also not unusual to find reports that include statistical comparisons of multiple variables. Manuscript reviewers should recommend avoiding unplanned multiple testing (Assmann et al. 2000) unrelated to the study hypothesis. Studies may have also been designed to test multiple hypotheses. Testing multiple hypotheses inflates Type I statistical error rate (Benjamini et al. 2001; Benjamini and Hochberg 1995; Glickman et al. 2014; Ioannidis 2018) (inflated α = 1 − (1 − α)^N^, *N* = number of hypotheses tested) (Rothman 1990). Multiple comparison correction intends to circumvent the problem that as the number of tests increases, so does the likelihood of a Type I statistical error (Benjamini et al. 2001; Benjamini and Hochberg 1995; Lee and Lee 2018).

On the other hand, multiple comparisons correction can increase the Type II statistical error rate (Ioannidis 2018). If no statistical correction is implemented for multiple comparisons, it is best practice to report all individual *p* values and confidence intervals and acknowledge that no mathematical correction was made for multiple comparisons. When conducting multiple comparisons, researchers frequently attempt to control for the increased risk of Type I errors by adjusting their alpha or significance threshold levels (Bender and Lange 2001; Ioannidis 2018).

There are two common approaches for controlling Type I statistical error when testing multiple hypotheses: control the false discovery rate and control the Type I statistical error rate for the family of comparisons. The false discovery rate is the probability that a null hypothesis is true, given that the null hypothesis has been rejected. The algorithms (e.g., the Benjamini and Hochberg, Benjamini, Krieger, and Yekutieli or Benjamini and Yekutieli, etc.) used for deciding which *p* values are small enough to be a “discovery” need to be reported (Benjamini et al. 2001; Benjamini and Hochberg 1995; Lee and Lee 2018, Benjamini et al. 2006). Tests for controlling the Type I statistical error rate for the family of comparisons include Dunnett's (used when comparing treated groups with the same control group), Tukey's test (used to make all possible pairwise comparisons) (Tukey 1949), Scheffe's test (may be used to make more complex comparisons than pairwise comparisons among means) (Scheffe 1959), and Bonferroni (Bonferoni 1936) or Šídák (Šidák 1967) (for a preplanned set of means to compare). Dunn's test is used for nonparametric data (Dunn 1964). Considering the multiple options for comparative analysis, it is important to report the implemented multicomparison testing strategy to favor reproducibility in data analysis (Bender and Lange 2001).

## 4 Other Considerations

### 4.1 Data Analysis Interpretation

Authors and reviewers should ensure that correct language involving frequentist significance testing is used. *P‐*values and significance tests have been misinterpreted and misused in biomedical research (Amrhein et al. 2019, Baker 2016a). The readers of this paper are encouraged to review American Statistical Association recommendations for the correct use and interpretation of *p* value and significance level (ASA 2016).

In frequentist inferential analysis, researchers must avoid an overinterpretating *p* values and bear in mind at least two fundamental limitations of significance testing. (i) The fallacy of classical inference, which states that with sufficient power, the null hypotheses can be rejected with a trivial effect (Barnett and Mathisen 1997; Halsey et al. 2015; Silva‐Ayçaguer et al. 2010). *P‐*values do not convey any information about the effect size or the clinical importance of the observed effect (Halsey et al. 2015). (ii) Significance level was conceptualized as a continuous variable to aid judgment but not as an absolute index of the truth about the evidence against the null hypothesis. Reporting *p* values along effect size confidence intervals reduces the overinterpretation of the *p* values (Amrhein et al. 2019).

For every conclusion, there is evidence for, evidence against, and uncertainty about how far it can be generalized. *P‐*values from the statistical analysis larger than the predefined significance level only indicate insufficient evidence against the null hypothesis (ASA 2016). Statistically, nonsignificant results do not *prove* the null hypothesis and do not imply a lack of treatment effects or equivalence. Regrettably, countless published reports ignore this basic concept (Amrhein et al. 2019).

A negative result (e.g., lack of pharmacological effect) can be the result of several reasons: (1) the real difference between groups is less than the hypothesized amount during the sample size calculation, (2) there is no difference between groups, (3) the variance of the observed data was greater than anticipated, (4) there were confounding factors in the conduct of the study or analysis of the data that led to a smaller difference than exists, and (5) Type II statistical error.

Authors, reviewers, and editors should consider that a positive outcome (hence, rejecting the null hypothesis) from a single study could simply be a Type I statistical error. Study replications can rule out the chance of incurring a Type I statistical error.

## 5 Conclusion

The statistical reporting needs to be sufficiently detailed to ensure the integrity of scientific studies. The level of detail should enable a qualified statistician to accurately repeat the analysis using the manuscript, study data set, and any supplemental material. In some cases, the detail required to describe statistical procedures fully may exceed the space constraints of a standard research manuscript. To address this situation, authors are encouraged to utilize a supplemental section to comprehensively explain procedures not covered in the main text due to space limitations. The data, code, and other interoperable and reusable files could be posted on a public and persistent website (the FAIR principles—Wilkinson et al. 2016).

The quality of scientific reports, including the thoroughness of reports' statistical sections, heavily depends on peer reviewers with varying levels of statistical expertise. Peer review processes do not always guarantee good reporting practices. Incomplete statistical reporting resulting from unintentional oversights and lack of knowledge hinders the data analysis of future studies. Identifying missing information in a scientific manuscript is generally more challenging than detecting included but incorrect information. Implementation of guidelines for reporting preclinical research, such as the ARRIVE guidelines (Kilkenny et al. 2010; Percie du Sert et al. 2020) and checklists at manuscript submission, helps improve reporting and reduce inadvertent oversights during the elaboration and review process of scientific reports (Curtis et al. 2022; Plint et al. 2006).

This review summarizes a series of critical statistical reporting elements (Table 3) that can help authors write complete reports, assist in triaging manuscripts early in the submission process, and ease the reviewer's workload. In the not‐too‐far future, artificial intelligence technologies can easily aid editorial tasks (http://www.statreviewer.com/) and check manuscripts against standardized statistical reporting guidelines (statcheck // web (shinyapps.io)).

**TABLE 3:** Statistical Reporting Protocol.

PROMPT	RECOMMENDATION
**Experimental design**	
1	*Provide a statement of the research question, including:* population and subpopulations of interest. Identify experimental units (e.g., individual vs. pen of animals), describe sampling and treatment design, blocking strategies, covariates, etc.
2	*Explain the methods used for sample size calculation*. Include the effect size, the source, and a justification for its use, including its biological/clinical relevance and the estimate of dispersion used. Clarify whether the calculation accounted for any attrition/censoring, truncation, and/or exclusion of experimental units. List the software(s) used.
3	*Randomization* Indicate whether the experimental units were randomized, the type and methodology of randomization, and the allocation ratio.
**Statistical strategy**	
4	*Reporting data handling* Identify the outcome variable(s) included in the analysis. Describe the statistical hypothesis (e.g., the μ of treatment A equals the μ of treatment B). Summarize the data for each variable with the appropriate descriptive statistics. Report the total sample and group sizes for each analysis. Report any data impacted by changes from the original intentions of the protocol. This includes dropouts of experimental units and the deletion or replacement of observations. If there were experimental unit dropouts, state whether they were replaced. If dropouts were replaced, explain how they were randomized. Report whether the data included outliers and how those were identified and handled. Report if the data excluded data points and the reason(s) for excluding data points. Report the values of the removed data. If any data points are imputed to replace missing data, explain the imputation process. Report if the data from unequal‐size groups was corrected (e.g., Welch's correction). Report how the data were transformed or scaled. If data were transformed, it should be back‐transformed to be expressed on the original scale of the measurement. Report whether the statistical analysis was masked (blinded statistician).
5	*Reporting statistical models* Report the statistical hypothesis Report the statistical test(s) and model implemented for each outcome variable assessed. Report model specifications and a summary model specification (e.g., goodness‐of‐fit) test. When applicable, mention the one‐sidedness of the test if a one‐sided test is used and explain precisely which model was fitted to the data and whether (and how) data were weighted (e.g., for a curve using nonlinear regression). For multilinear regression analysis, The strategies for the selection of explanatory variables (e.g., backward and forward selection) in model building should be presented as part of the model selection. V For Linear mixed models: Report the fixed and random terms of the final model Indicate the covariance structure included in the final model VI For Bayesian analysis: Explain the model: (a) Explain the dependent variables and independent variables. (b) Explain the likelihood function and all the parameters. (c) Explain and justify the prior distribution of the parameters in the model. (d) Include mathematical or computer code of the likelihood and prior. (e) Provide a prior predictive check. Report computation details: (a) Markov chain Monte Carlo chain convergence. (b) Markov chain Monte Carlo chain resolution. Describe the posterior distribution, posterior predictive check, state whether density‐based values or quantile‐based values are used, and the credible interval. (c) Bayesian Factor and posterior model probabilities. Report decisions (if any) and their criteria; (a) Loss function. (b) Region of practical equivalence limits. (c) Bayes factor, decision threshold, and justify the decision threshold for the posterior model probability and the minimum prior model probability that would make the posterior model probability exceed the decision threshold. Report sensitivity analysis (a) For broad priors. (b) For informed priors. (c) For default priors. (d) Bayes factors and model probabilities. If making decisions, report whether decisions change under different priors. For Bayes factors, report changes in the minimum prior model probability needed to achieve decisive posterior model probability. Report the computer code.
6	*Report the assumptions of the statistical tests*. How were they tested, and did they report whether the assumptions were met? Describe what was done if the statistical assumptions were unmet (a list of statistical assumptions and recommended tests is listed in Table 2).
7	*Report the strategy implemented to control type I statistical error inflation* (e.g., *false discovery rate*) *in the case of multi‐comparison testing*. If type I statistical error was controlled, report the adjusted *p* values. It is good statistical practice to inform whether all comparisons were planned. Authors should explicitly state when no corrections for multiple comparisons have been made.
8	*Report the significance level*. For any given hypothesis, the authors should provide the test used to obtain the corresponding *p* value.
9	*List the statistical software package*(*s*) Include any specific added packages or plugins used in the analysis.
**Statistical results and interpretation**	
10	Summarize data results Report numbers with an appropriate degree of precision. Summarize approximately normally distributed data with means and standard deviations. Otherwise, median and range should be used. Standard error of the means should not be mistakenly used in the attempt to summarize the data set variability. Unbiased effect size estimates should be reported with their precision estimates, such as confidence intervals.
11	Avoid misinterpreting or overstating significant statistical differences and ensure congruency in axis [scientific question—statistical question—statistical strategy—study results—study conclusion]. Authors should use correct language that reflects the meaning of a frequentist significance test.

*Note:* The author confirm that the ethical policies of the journal, as noted on the journal's author guidelines page, have been adhered to. No ethical approval was required as no animals were used.

The main purpose of this study is to serve as a reporting guideline rather than just a statistical tutorial. The content of this report will continually evolve and adapt to meet new requirements and the evolving submission landscape, aiming to foster reproducible veterinary pharmacology science.

## Conflicts of Interest

The author declares no conflicts of interest.

## Data Availability

Data sharing is not applicable to this article as no new data were created or analyzed in this study.
